# Parents' reasons to vaccinate their children aged 5–11 years against COVID-19 in Italy

**DOI:** 10.3389/fmed.2022.949693

**Published:** 2022-08-02

**Authors:** Annalisa Napoli, Grazia Miraglia del Giudice, Francesco Corea, Lucio Folcarelli, Italo Francesco Angelillo

**Affiliations:** ^1^Department of Experimental Medicine, University of Campania “Luigi Vanvitelli”, Naples, Italy; ^2^Department of Public Health and Laboratory Services, Teaching Hospital of the University of Campania “Luigi Vanvitelli”, Naples, Italy

**Keywords:** children, COVID-19, Italy, parents, reasons, vaccination

## Abstract

**Objectives:**

The aims of this cross-sectional study were to investigate why parents decide to vaccinate, as well as the determinants, their children aged 5–11 years against COVID-19 in Italy.

**Methods:**

The survey was conducted from January through May 2022. All parents/guardians who came in randomly selected days to immunization centers for the administration of the first dose of the COVID-19 vaccine to their child were asked to complete a questionnaire about socio-demographic characteristics, attitudes toward COVID-19 infection and vaccination, reason(s) regarding their decision to vaccinate their child, and source(s) of information.

**Results:**

A total of 358 questionnaires were collected. Parent's perception that COVID-19 is a severe illness for the child, assessed using a 10-point Likert scale, was 7.5. The overall mean scores of the risk perception for their child of having the COVID-19 before and after the vaccination were 8.1 and 6.3. A significantly higher parents' level of risk perception for their child of having the COVID-19 after the vaccination has been observed among those not having a university degree, those with the child having at least one chronic medical condition, and those who perceived that COVID-19 is a severe illness for the child. The mean value of respondent trust in the information provided by the pediatricians on a 10-point scale Likert type was 7.6. Female, not having a university degree, higher perception that COVID-19 is a severe disease, not having received information about the vaccination from pediatricians, and needing information had a significantly higher concern of side effects after the vaccination. The most common reasons for vaccinating their children included wanting to protect the child against COVID-19, to attend the school with less risk, to prevent the transmission to family members, and to practice sport and other activities with less risks. Participants with a university degree were more likely to have vaccinated their child for attending the school and practicing sport and other activities with less risks.

**Conclusions:**

More publicity should be promoted among parents of children aged 5–11 years which would increase the coverage rates and thus lower the transmission of SARS-CoV-2 and reduce the occurrence of COVID-19.

## Introduction

As of the end of April 2022, in Italy the number of reported confirmed cases of COVID-19 exceeded 16.8 million with more than 16 thousand deaths caused by the SARS-CoV-2 (1). It is well-known that children had similar incidence rates of SARS-CoV-2 infection compared with adults (2) and that the vaccination among this group is essential to reduce infection and transmission to the susceptible person.

On December 1, 2021, the Italian Medicines Agency (AIFA) has authorized the administration of two 10-μg doses of the Pfizer-BioNTech BNT162b2 mRNA COVID-19 vaccine 21 days apart to children ages 5 through 11 years (3). On December 7, 2021, the Ministry of Health announced that this age group who do not have contraindications to the vaccine could receive this free complete series (4). On December 16, 2021, the national vaccination campaign started in almost all regions of the country throughout the community vaccination centers. However, despite the high frequency of the disease and the safe and real-world data on vaccine effectiveness among children aged 5 to 11 years of age in reducing symptomatic disease, hospitalizations, and deaths (5–8), as of May 17, 2022, in Italy the vaccination rates were low in this group since only 37.9% had received one dose and 34.5% had received their second dose (9).

Parents' COVID-19 vaccination hesitancy or likelihood of their children getting this vaccine has been widely debated (10–13). However, until now very few studies have focused on parents' reasons to have their child 5–11-year-olds vaccinated and the associated factors and understanding this issue is essential in planning effective measures for increasing vaccination uptake and avoiding fueling vaccine hesitancy. Therefore, the present cross-sectional study attempts to investigate why parents decide to vaccinate, as well as the determinants, their children aged 5–11 years against COVID-19 in Italy.

## Materials and methods

### Setting and target population

Data were collected as a part of a larger project on perceptions and behaviors toward COVID-19 vaccination among different groups of people living in Southern Italy (10, 11, 14–18). This cross-sectional survey was conducted from January through May 2022 in two randomly selected immunization centers located in the geographic area of Naples, Southern part of Italy. All parents/guardians aged ≥ 18 years of children 5–11 years of age who came in randomly selected days to the immunization centers for the administration of the first dose of the COVID-19 vaccine to their child were approached in the waiting rooms and asked about their interest in participating in the study.

The sample size was calculated by using single population proportion formula, assuming that 25% of the respondents vaccinated their child for attending the school and practicing sport and other activities with less risks, with a margin of error of 5%, a confidence interval of 95%, and an expected response rate estimated of 85%. This gives the final sample size of 321 participants.

### Study procedures

The protocol and the informed consent of the study were approved by the Ethics Committee of the Teaching Hospital of the University of Campania “Luigi Vanvitelli”. Before participation in the study, well-trained research team members in conducting surveys with self-administered questionnaires approached each parent/guardian and, after introducing themselves, asked if he/she would be interested in participating in this study. They were fully informed about its purpose and significance, that the participation was completely voluntary, that the questionnaire was anonymous and will not include any identifiers or personal information of the participants, that the information will be kept private and confidential, that they could stop completing the questionnaire at any stage, and the information will only be used for scientific research purposes.

The research team members collected the study questionnaires from parents/guardians once they were filled. Informed written consent was obtained from participants before the questionnaire was administered to them. No incentives or rewards were offered to participants as compensation for their time.

### Survey instrument

The questionnaire was developed on the basis of previous instruments used in similar surveys carried out by some of us to evaluate parental and/or individual COVID-19 vaccination acceptability enrolling different populations (10, 11, 14–18). A total of 10 non-selected individuals were asked about the questionnaire's clarity, wording, and as well as whether any of the questions were difficult to comprehend, before disseminating the final questionnaire to the research population. Participants in the pilot study were not included in the final study sample.

The questionnaire was self-administered and took approximately 5 min to complete. The questionnaire was organized into three parts. In the first part, questions were asked about parents' socio-demographic characteristics (i.e., gender, age, employment status, educational level, marital status, number of children in home, having been infected with SARS-CoV-2) and children's characteristics (i.e., age and gender). In the second part, attitudes toward the COVID-19 infection (risk perception for their child of having the COVID-19 before and after the vaccination, perceived severity of COVID-19) and attitudes toward the COVID-19 vaccination (concern about serious adverse effects from COVID-19 vaccine for their child, trust in the information provided by the pediatricians). These questions were collected on a 10-point Likert scale, ranging from 1 representing not at all to 10 representing at all. In the third part, the parents were asked whether they had received and from whom the recommendation for COVID-19 vaccine for their child, the reason(s) regarding their decision to vaccinate their child, and also whether they had any doubts regarding the COVID-19 vaccine for their child and the reason(s) associated with. In the response with 8 options, respondents could select all that apply. Finally, the participants were asked which source(s) of information about COVID-19 vaccination for children 5–11-year-olds they had used, including mass media, Internet, pediatrician, physician (other than pediatrician), friends, social network, official government organizations, and scientific journals, and they were asked to select all responses that applied. Respondents were also asked whether they would like to obtain additional information on this topic in the future.

### Statistical analysis

First, descriptive statistics including relative frequency, mean, and standard deviation were used to summarize the personal characteristics of respondents and their child. Second, bivariate associations between each variable and the continuous or dichotomous outcome have been tested using when appropriate the chi-square test or the Student's *t*-test. Variables associated in the bivariate analysis with a *p*-value ≤ 0.25 were entered into the multivariate linear and logistic regression models. Third, multivariate linear and logistic regression models were employed to identify the determinants of the following dependents variables: risk perception for their child of having the COVID-19 after the vaccination (continuous) (Model 1); concern that their child can report side effects after receiving the vaccination (continuous) (Model 2); and having vaccinated their child for attending the school and practicing sport and other activities with less risks (no = 0; yes = 1) (Model 3). The following independent variables have been selected because potentially related to all dependents variables: gender of the child being vaccinated (male = 0; female = 1), age of the child being vaccinated (continuous), child being vaccinated with at least one chronic condition (no = 0; yes = 1), respondent's age in years (continuous), gender (male = 0; female = 1), baccalaureate/graduate degree (no = 0; yes = 1), having other children in home (no = 0; yes = 1), having received the COVID-19 vaccine (no = 0; yes = 1), having contracted SARS-CoV-2 (no = 0; yes = 1), at least one parent/cohabitant partner who contracted SARS-CoV-2 (no = 0; yes = 1), at least one parent/cohabitant partner who received the COVID-19 vaccine (no = 0; yes = 1), believing that COVID-19 is a severe illness (continuous), having being recommended to vaccinate their child (no = 0; yes = 1), having received information on COVID-19 vaccination from pediatrician (no = 0; yes = 1), and need of additional information on COVID-19 vaccination (no = 0; yes = 1). The variable marital status (unmarried = 0; married/cohabited with a partner = 1) was included in Models 2 and 3; the variables at least one parent/guardian being a healthcare worker (no = 0; yes = 1) and having trust in the information received from the pediatrician (continuous) were added in Models 1 and 2; and the variables risk perception for their child of having COVID-19 before the vaccination (continuous), risk perception for their child of having COVID-19 after the vaccination (continuous), concern about serious adverse effects from COVID-19 vaccine for their child (continuous), and having doubts regarding the COVID-19 vaccine for their child (no = 0; yes = 1) were included in Model 3.

A stepwise method was used to retain or to exclude in the final multivariate models the variables with a threshold of *p* = 0.2 and *p* = 0.4, respectively. In the multivariate logistic regression model, odds ratio (OR) with a 95% confidence interval (CI) was computed, whereas in the linear regression models standardized regression coefficient (ß) was used. All statistical tests were two-tailed and *p-*values equal to or <0.05 were considered to be statistically significant. All statistical analyses were conducted in STATA software version 15.1.

## Results

Of the 370 parents/guardians who were randomly selected only 12 refused to participate in the survey and 358 agreed for a response rate of 96.8%. The socio-demographic and general characteristics of the respondents are presented in Table 1. The mean age was 41.7 years, 70.1% was female, more than three-quarters were married, for 34.4% the highest level of education was a university degree, 66.2% was employed, less than one-third had one child, almost all had been vaccinated against COVID-19, and 21.6% have had at least one cohabitant who have contracted SARS-CoV-2.

**Table 1:** Socio-demographic and general characteristics of the study population.

**Characteristics**	***N***	**%**
**Parent**		
**Age, years**	41.7 ± 6.5 (23-60)
**Gender**		
Female	251	70.1
Male	107	29.9
**Marital status**		
Married/cohabited with a partner	323	90.5
Unmarried/separated/divorced/widowed	35	9.5
**Educational level**		
High school degree or less	223	62
Baccalaureate/graduate degree	135	38
**Professional status**		
Employed	237	66.2
Unemployed	121	33.8
**Partner's professional status**		
Employed	277	80.5
Unemployed	81	19.5
**Number of children**		
1	107	29.9
> 1	251	70.1
**Having been infected by SARS-CoV-2**		
No	284	79.3
Yes	74	20.7
**Having at least one parent/cohabitant partner who had been infected by SARS-CoV-2**		
No	280	78.4
Yes	77	21.6
**Vaccinated against COVID-19**		
No	8	2.2
Yes	350	97.8
**At least an adult cohabitant vaccinated against COVID-19**		
No	12	3.3
Yes	346	96.7
**At least another son/daughter vaccinated against COVID-19**		
No	155	43.3
Yes	203	56.7
**Vaccinated child**		
**Age, years**	8.4 ± 2
**Gender**		
Female	161	45.5
Male	193	54.5
**Birth order**		
First	211	59.1
Second	121	33.9
≥Third	25	7
**Underlying chronic medical conditions**		
No	268	74.9
Yes	90	25.1
**Parent's rate health status**	9.3 ± 1.1 (5–10)

*Mean ± Standard deviation (range).

The perception that COVID-19 is a severe illness for the child, assessed using a 10-point Likert scale, was generally high among the respondents with a mean value of 7.5 and about a third reported a value of 10 (30.2%). Table 2 reported the results of the multivariable linear and logistic regression analysis examined the independent association of several determinants and the different outcomes of interest. Multivariable linear regression analysis showed that a significantly higher parents' level of risk perception for their child of having the COVID-19 after the vaccination has been observed among those who did not have a university degree, those with the child having at least one chronic medical condition, and those who perceived that COVID-19 is a severe illness for the child (Model 1). Regarding the risk perception for their child of having the COVID-19, the overall mean score was, respectively 8.1 before and 6.3 after the vaccination. Overall, 43.5 and 19.8% respondents had the higher score giving a rating of 10. Only 14.7% of participants expressed the higher concern, with a response of 10 on a 10-point Likert type scale, about serious adverse effects from COVID-19 vaccine use for their child with an overall mean value of 6. In a multivariable linear regression model examining the association of multiple factors with the concern of reporting side effects after receiving the vaccination it has been observed that female gender, not having a university degree, higher perception that COVID-19 is a severe disease, not having received information about the vaccination for their children from pediatricians, and needing additional information about COVID-19 vaccination for children 5–11-year-olds were associated with a higher level of concern (Model 2 in Table 2). The mean value of respondent trust in the information provided by the pediatricians on a 10-point scale Likert type was 7.6, but only less than one-third (29.9%) expressed the higher level of trust. More than half of the parents/guardians (58.9%) reported receiving recommendation for COVID-19 vaccine for their child and the health care providers in the pediatric primary care setting were those who most frequently make the recommendation (75.4%). The most common reasons the parents reported for vaccinating their children against COVID-19 included wanting to protect the child against COVID-19 (86.9%), to attend the school with less risks (33.8%), to prevent the disease transmission to other family members (27.4%), and to practice sport and other activities with less risks (17%). Overall, only 12.6% of the parents vaccinated their child for attending the school and practicing sport and other activities with less risks. The results of a multivariable logistic regression model showed that participants with a university degree were more than two times (OR = 2.32; 95% CI 1.21–4.46) more likely to have vaccinated their child for this reason than those with a lower level of education (Model 3 in Table 2). In addition, the respondents' worry about the adverse effects of the vaccination (59.1%) and the feeling that they did not have enough information regarding the child's vaccination (38.5%) were their main doubts before the vaccination.

**Table 2:** Determinants of the different outcomes of interest using linear and logistic regression analysis.

**Variable**	**Coeff**.	**SE**	***t***	***p***
**Model 1**. Parents' risk perception for their child of having the COVID-19 after the vaccination				
*F*_(8, 29)_ = 8.22, *p* < 0.0001, *R*^2^ = 16.7%, adjusted *R*^2^ = 14.6%				
Believing that COVID-19 is a severe illness	0.27	0.05	5.11	<0.001
Not having a baccalaureate/graduate degree	−0.92	0.27	−3.40	0.001
Child vaccinated with at least one chronic condition	0.73	0.31	2.34	0.02
Female	0.49	0.28	1.70	0.089
Female child vaccinated	0.41	0.26	1.56	0.12
At least one parent/cohabitant partner who contracted SARS-CoV-2	0.44	0.32	1.39	0.164
Need of additional information on COVID-19 vaccination	0.31	0.29	1.10	0.273
Neither parent/guardian being a healthcare worker	−0.49	0.50	−0.98	0.329
**Model 2**. Parents' concern that their child can report side effects after receiving the vaccination				
*F*_(11, 327)_ = 6.68, *p* < 0.0001, *R*^2^ = 18.3%, adjusted *R*^2^ = 15.6%				
Female	1.30	0.30	4.33	<0.001
Believing that COVID-19 is a severe illness	0.20	0.05	3.45	0.001
Not having received information on COVID-19 vaccination from pediatricians	−0.80	0.28	−2.81	0.005
Need of additional information on COVID-19 vaccination	0.65	0.30	2.18	0.03
Not having a baccalaureate/graduate degree	−0.60	0.28	−2.14	0.033
Child vaccinated with at least one chronic condition	0.65	0.33	1.94	0.053
Having been recommended to vaccinate their child	0.50	0.28	1.77	0.077
At least one parent/cohabitant partner who received the COVID-19 vaccine	1.31	0.77	1.70	0.089
Female child vaccinated	0.44	0.27	1.63	0.104
Neither parent/guardian being a healthcare worker	−0.55	0.54	−1.02	0.307
At least one parent/cohabitant partner who contracted SARS-CoV-2	0.33	0.33	1.02	0.31
	**OR**	**SE**	**95% CI**	***p***
**Model 3**. Parents having vaccinated their child for attending the school and practicing sport and other activities with less risks				
Log likelihood = −127.56, χ^2^ = 13.72 (4 df), *p* = 0.0083, adjusted *R*^2^ = 5.1%				
Having a baccalaureate/graduate degree	2.32	0.77	1.21–4.46	0.012
Having doubts regarding the COVID-19 vaccine for their child	1.81	0.63	0.91–3.60	0.089
Not having parent/cohabitant partner who contracted SARS-CoV-2	0.56	0.26	0.23–1.41	0.221
Lower risk perception for their child of having COVID-19 after the vaccination	0.93	0.06	0.82–1.06	0.285

Most of the sample (87.2%) had learned about COVID-19 vaccination for children 5–11-year-olds. Almost half of the respondents said that their child's healthcare provider (45.5%) was the most important source of information about vaccines followed by the physicians (36.3%). Additional sources were government agencies (27.4%), mass media (18.7%), and Internet (16.5%). One-third (31.7%) reported that they want to obtain more information regarding vaccination.

## Discussion

The results of the present survey provide a detailed description that contributes to an understanding of the reasons of parents for deciding to vaccinate against COVID-19 their children aged 5–11 years, as well as the determinants. These results can provide a useful guidance to decision makers and healthcare workers on approaches to take when designing interventions in this field.

It is interesting to note that several reasons have been reported by parents in support of the decision to vaccinate their children against COVID-19. The vast majority of the respondents reported that they vaccinated their children because they were to protect the child from the disease, whereas additional reasons were aligned as feeling that vaccination was a means of attending the school with less risks and protecting the family members. These reasons are confirmed by several previous studies on parents' willingness to accept the COVID-19 vaccination for their children (10, 19, 20). Moreover, the present survey demonstrates that parents' concerns about adverse effects of the vaccination were the biggest doubt before the vaccination. This is consistent with the findings of several previous studies, local and abroad, also regarding the willingness or hesitancy of the vaccination (11–13, 21, 22). However, this finding is surprising particularly because it has been observed worldwide that the most reported adverse events of the COVID-19 vaccination in children and adolescents, particularly with the mRNA vaccines, were mild in severity and short in duration (23, 24). These results highlight the responsibility of policymakers in addressing the critical issue of educating the public on the safety of the vaccination.

It has been observed that the physicians were the highly preferred source of information among parents/guardians about the COVID-19 vaccination for their children. Compared to parents who heard about COVID-19 vaccine through physicians, concern about side effects of the vaccine has been more likely to be reported by those who said that they did not acquire information from this source. This is consistent with the findings in the literature showing the important role of these professionals in providing comprehensive and objective information on this issue and for increasing the confidence of the vaccine (10, 18, 25–29). This finding also underlined the fact that physicians play a larger role in how healthcare is provided, and they have a unique opportunity to ensure that parents understand the benefits, safety, and efficacy of the COVID-19 vaccine and the importance of getting the vaccine as an essential preventive health care. Discussions with physicians, as with other health care professionals, are also important for providing education and parents should be able to have open conversations with them and to address their concerns and questions. Such conversations about the opportunities for vaccination would enable parents to work together with these providers to consider how best to protect their children. Moreover, it should be underlined that only half of the parents/guardians had received recommendation for COVID-19 vaccine for their child and the health care providers in the pediatric primary care setting were those who most frequently make the recommendation. This finding could be used by healthcare providers to deliver appropriate messages about risks and benefits to parents and this is also confirmed by the fact that one-third of the respondents had indicated an interest in receiving additional information. Another interesting finding of the present study was the small proportion of respondents that had used Internet as a source of information. This result is comfortable due to the anti-vaccination messaging and the spread of inaccurate and misleading public health information around COVID-19 and its vaccines circulating online since the beginning of the pandemic (30–32).

Multivariate regression analysis revealed that a number of socio-demographic and general characteristics of the respondents and of the vaccinated child were associated with the different outcomes of interest. It has been identified that gender and educational level have a significant impact. Indeed, females and those with a lower educational level had a higher concern of side effects of the vaccination and this reflects the general trend in access to COVID-19 vaccine disparities that has been observed in several previous studies (33–37). The reason by which females are more concerned may be explained by the fact that in Italy they were more affected than men although male presented a higher risk of death (38). With regard to the results of the educational level, a possible explanation is that parents with a higher level are able to get more information easier than the general population that makes them advantageous over part of the other societies. Similar explanation for significant impact of the finding with parents less educated that were more likely to perceive a higher level of risk perception for their child of having the COVID-19 after the vaccination. Further, it has been observed that the health condition of the vaccinated child has also a significant impact, with parents of the child with at least a chronic medical condition had a higher level of risk perception for their child of contracting the SARS-CoV-2 after the vaccination. This may suggest that these parents still perceived for their child a higher degree of vulnerability than those without a chronic medical condition. Finally, the finding that individuals who perceive COVID-19 to be a severe disease are anxious about contracting it for their child is consistent with the literature in other countries (39–41).

The findings should be interpreted in the light of some potential methodological limitations derived from the nature of this study. Firstly, because of the nature of the cross-sectional study method that has been used the identified associations may be difficult to interpret since it is difficult to draw conclusions regarding the direct causal inferences and the direction of causality. Secondly, since the participants were selected in one city, the generalization of the results to other geographic areas of Italy should be made with caution, but they could reflect the population with similar socio-demographic characteristics. Thirdly, as in most surveys, parent-reported information is subjective and may be affected by social desirability. However, since COVID-19 can be a sensitive and important issue for the majority of respondents, it is unlikely that social desirability bias resulted, for example, in under-reporting of their own or their familiar having been infected with SARS-CoV-2. We are likely to expect that respondents' reporting of their experience should be reasonably accurate, as having the infection is a rather an important event and therefore likely to be memorable.

In conclusion, parents of children aged 5–11 years exhibited, although vaccinated, concerns about side effects and a lower use of healthcare workers as a source of information about vaccines. This finding shows the essential role played by the physicians to provide adequate information to the parents about benefits, safety, and efficacy of the COVID-19 vaccine. In this context it is also interesting that only half of the participants had received recommendation for COVID-19 vaccine for their child. Since the COVID-19 vaccination rate is low in this age group, this study underlines the need to improve publicity among parents of children aged 5–11 years in order to increase the rates and thus lower the transmission of SARS-CoV-2 and reduce the occurrence of COVID-19.

## Data availability statement

The raw data supporting the conclusions of this article will be made available by the authors, without undue reservation.

## Ethics statement

The studies involving human participants were reviewed and approved by Ethics Committee of the Teaching Hospital of the University of Campania “Luigi Vanvitelli”. Written informed consent to participate in this study was provided by the participants' legal guardian/next of kin.

## Author contributions

AN, GMG, FC, and LF participated in the design of the study, contributed to the data collection, the data analysis, and interpretation. IFA the principal investigator, designed the study, was responsible for the statistical analysis and interpretation, and wrote the article. All authors have read and approved the final version of the manuscript.

## Funding

This work was supported by a grant of the Regione Campania (Executive decree n.75/2017 Strategic and nationally relevance objectives indicated in the National Health Plan. FSN 2014, 2015, 2016).

## Conflict of interest

The authors declare that the research was conducted in the absence of any commercial or financial relationships that could be construed as a potential conflict of interest.

## Publisher's note

All claims expressed in this article are solely those of the authors and do not necessarily represent those of their affiliated organizations, or those of the publisher, the editors and the reviewers. Any product that may be evaluated in this article, or claim that may be made by its manufacturer, is not guaranteed or endorsed by the publisher.
